# Timing and Intensity of Early Intervention Service Use and Outcomes Among a Safety-Net Population of Children

**DOI:** 10.1001/jamanetworkopen.2018.7529

**Published:** 2019-01-25

**Authors:** Beth M. McManus, Zachary Richardson, Margaret Schenkman, Natalie Murphy, Elaine H. Morrato

**Affiliations:** 1 Department of Health Systems, Management, and Policy, Colorado School of Public Health, Aurora; 2 Physical Therapy Program, University of Colorado School of Medicine, Aurora

## Abstract

**Importance:**

Federal per-child early intervention (EI) appropriations have declined, while accountability for improving children’s development and function has increased. It is critical to understand high-value EI services and systems.

**Objective:**

To examine EI service timeliness and intensity, and the association between service intensity and outcomes.

**Design, Setting, and Participants:**

This secondary data analysis cohort study linked pediatric primary care electronic health records and EI program records from October 1, 2014, to September 30, 2016. Sample children from a large, urban safety-net health system and EI program who were younger than 35 months with a developmental disability or delay were examined. Data analysis was conducted from December 15, 2017, to May 15, 2018.

**Exposures:**

The study included measures of condition type and severity, race and ethnicity, family income, insurance type, sex, birth weight, and language.

**Main Outcomes and Measures:**

The timeliness of EI (days from referral to EI care plan), service intensity (hours per month) overall and for core EI services (physical, occupational, speech therapy, and developmental intervention), and change in function (measured on a 13-point scale). Adjusted quantile median regression estimated timeliness and intensity. Adjusted linear regression estimated change in function.

**Results:**

Of the 722 children who received an EI care plan (median [interquartile range] time to receive EI care plan, 56.0 [1.0-111.0] days) 457 (63.3%) were male, 447 (62.0%) were younger than 12 months, 207 (28.7%) were 12 to 24 months, and 68 (9.3%) were 25 to 35 months. A total of 663 children (91.8%) had a household income of less than $20 000 annually; 305 (43%) of the sample children received an EI care plan within the 45-day deadline. Median (interquartile range) for EI intensity was 2.7 (2.3-3.6) hours per month. Children living above the federal poverty threshold received greater occupational therapy intensity (b, 1.9; 95% CI, 0.9-3.0). Greater clinical severity was associated with more timely receipt of an EI care plan. Compared with infants, 2-year-old children received a care plan almost 2 months sooner (b, −52.0; 95% CI, −79.7 to −24.3). An additional hour per month of EI service was associated with a 3-point functional gain (b, 3.0; 95% CI, 1.5-5.9) among children with complete outcomes information (n = 448).

**Conclusions and Relevance:**

In this study, greater EI service intensity was associated with better functional gains, yet most children in the study received delayed care and/or low service intensity. Clinical and EI record linkages could serve as a framework for improving EI processes.

## Key Points

**Question:**

What is the timeliness and service intensity of children enrolled in early intervention, and is service intensity associated with greater gains in function?

**Findings:**

In this cohort study, more than half of the study children received delayed early intervention access and most received low-intensity early intervention services. An additional hour per month of early intervention services was associated with a 3-point gain in functional outcomes.

**Meaning:**

Linking clinical and early intervention records could help integrate data to track early intervention–eligible children and improve early intervention timeliness, service intensity, and outcomes.

## Teaser

This cohort study examines the timeliness and intensity of early intervention services received and whether there is an association between early intervention service use and changes in children’s functional outcomes among a sample of early intervention–referred infants and toddlers with developmental delays and disabilities.

## Introduction

The Individuals with Disabilities Education Act provides funding to states to establish statewide early intervention (EI) systems of care to provide developmental and therapeutic services for infants and toddlers with developmental delays and disabilities.^1^

The benchmark for EI timeliness is driven by federal EI policy, which mandates that an EI care plan be written within 45 days of referral receipt.^1^ Previous research, conducted nearly a decade ago, suggests that children living in poor neighborhoods have less timely EI access.^2^ However, this research focused on service provider designation (not subject to the 45-day deadline) rather than care plan development. Thus, current patterns of EI timeliness in an era of fiscal constraints to support EI programming need to be evaluated.

The benchmark for optimal EI service intensity is less clear. While pediatric therapy intensity guidelines exist,^3,4^ these do not always translate to EI, which includes a clinically diverse population and different service delivery model. Yet, the first step to understanding optimal EI therapy intensity is to establish current EI service intensity and evaluate how this varies by clinical and social factors and relates to functional gains.

Moreover, there is a critical gap in the literature linking service use and functional outcomes. State EI programs are now required to report children’s function at EI entry and exit.^5^ These data have the potential to capture the extent to which EI enhances optimal outcomes, but to our knowledge have not been evaluated relative to EI service use intensity among EI-enrolled children.

In this study, EI timeliness, intensity, and functional outcomes were examined. This study sample includes EI referred children with developmental disabilities and delays who received primary care at a large, urban safety-net health system. This sample was chosen because very low-income families face greater EI access barriers. Moreover, we leveraged a unique opportunity to link pediatric primary care and EI electronic records. The aims of the study are to examine the association between developmental, clinical, and social characteristics and EI timeliness and service intensity and EI service intensity and outcomes among a low-income safety-net population of infants and toddlers with developmental disabilities and delays.

## Methods

### Sample

This cohort study was a secondary data analysis study of linked Denver Health and the Rocky Mountain Human Services Early Intervention Program (RMHS) EI records. Denver Health is a large safety-net health system in Metro Denver, Colorado, which serves approximately 50 000 low-income children annually. The Denver Health electronic records include children’s diagnoses and sociodemographics. The RMHS, located in Metro Denver, serves approximately 1000 families annually. The RMHS database includes children’s diagnoses, sociodemographics, and service use. Denver Health children are principally referred to the study EI program. This study was approved by the Colorado Multiple Institutional Review Board, which granted a waiver of consent for this secondary data analysis. This article was written in accordance with the Strengthening the Reporting of Observational Studies in Epidemiology (STROBE) reporting guideline for cohort studies.

Children were linked across the Denver Health and the RMHS EI databases using the child’s name and date of birth via a probabilistic matching algorithm (reclink2 algorithm in Stata). This algorithm allows for many-to-one matching between data sets where exact matching is either not feasible or inefficient. Observations were kept as acceptable matches if the resultant probability of an exact match was 85% or greater.

The study sample represents all children who received well-child care visits at Denver Health between October 1, 2014, and September 30, 2016, where the child initiated care at the RMHS. Sample children were younger than 35 months and had a diagnosed condition or developmental delay. Diagnosed conditions were identified from the Denver Health chronic condition database, which categorizes patients based on the following 4 chronic condition tiers: (1) no chronic health conditions; (2) mild severity (eg, vision or hearing loss, cleft lip/palate, and central nervous system anomaly); (3) moderate severity (eg, autism, intellectual disability, very low birth weight), and (4) greatest severity (eg, cerebral palsy, down syndrome, spina bifida). Colorado maintains a database of conditions associated with EI eligibility.^6^ Children whose conditions captured by Denver Health’s moderate and greatest severity groups and aligned with the Colorado EI eligibility database were included in the sample. Developmental delays were identified by the *International Classification of Diseases, Ninth Revision* code (ie, 315) from the child’s Denver Health record.

### Outcome Measures

The timeliness of EI was determined by the number of days from EI referral to creation of the EI care plan. The date of EI referral was captured from the health record and the care plan date was ascertained from the EI record.

Early intervention service intensity, overall and for each core EI discipline (physical therapy, occupational therapy, speech therapy [ST], developmental intervention [DI]), was captured from the EI record and determined by summing the total hours of all EI services received and dividing by the number of months the child was enrolled. Developmental intervention typically involves educational, play, and social interaction skills development.

Children’s EI outcomes following EI were estimated as the difference between exit and entry Child Outcomes Summary (COS) scores. Child Outcomes Summary score measures the child’s function in social relationships, cognition, and adaptive and/or self-care skills. Each domain score is a composite of parent and clinician report and formal assessment scaled by comparing the child’s function with that of typically developing, same-age peers, on a 7-point scale, from 1 representing very early skills (ie, child does not use any immediate foundational skills related to this outcome) to 7 representing all skills expected (ie, no concerns about the child’s function in this area).^7^ Currently, the study EI program does not have mechanisms in place to ensure routine collection of COS scores; therefore, there is not 100% compliance. To describe the association between service intensity and outcomes, our analysis was restricted to the 85% of children with complete COS information. The 2 groups were examined (those with and without complete COS information) to assess potential bias, but no differences were found between these groups of children on measured characteristics.

### Child Characteristics

Child’s race and ethnicity was categorized as white, non-Hispanic; black, non-Hispanic; Hispanic; and other race, non-Hispanic (includes Asian, Pacific Islander, and children reporting ≥1 race). Annual household income (<$20 000 or >$20 000) reflects the federal poverty threshold (FPL) for a family of 3.^8^ Infant birth weight categories (<1500, 1500-2500, and >2500 g) were included. As described earlier, children were categorized according to their condition severity group. Of note, children with developmental delays could (and often do) fall into either the no chronic condition or mild severity groups. Finally, children’s developmental condition was categorized as diagnosed condition (eg, cerebral palsy) or developmental delay. A measure of child’s sex was also included (male or female) and the primary language spoken at home (English vs a language other than English). Age at EI entry was categorized as less than 12 months, 12 to 24 months, or 25 to 35 months.

### Statistical Analysis

Analysis of descriptive statistics for each study variable was conducted. The EI outcome variables were found to be skewed, making median quantile model more appropriate in the estimating associations. We fit a series of adjusted median quantile regressions to first estimate EI timeliness and then EI service use controlling for child characteristics. For the service intensity models, overall service intensity was estimated first and then service intensity for each core EI service. For each parameter in the median quantile regression models, the β coefficient (95% CI) was presented.

To describe changes in children’s function following EI, we fit a series of adjusted linear regression models to estimate the association between change in each COS subscale score (ie, difference between EI exit and entry scores) and service intensity controlling for child characteristics. These models included the subsample of children (n = 448) with complete functional performance data (ie, COS scores at EI entry and exit). Given that service intensity data were skewed, service intensity variables were log transformed prior to fitting the regression models, then presented as results using retransformed data. All analyses were conducted in Stata, version 14.2 (StataCorp).

## Results

The sample included 722 children who received an EI care plan and of those, 525 (73%) initiated services, 448 (85%) of whom had complete functional outcomes information (eFigure in the Supplement). This represented 62% of the children who received an EI plan.

Sample children (N = 722) were predominantly Hispanic (532 [73.7%]) and had an annual household income below the FPL (<$20 000, 663 [91.8%]). Most of the sample children had a normal birth weight (>2.5 kg, 636 [88.1%]), developmental delay (633 [87.7%]) vs a diagnosed condition (89 [12.3%]), and no special health care need (472 [70.6%]), whereas 55 children (8%) had a condition categorized as most severe. Among children in the sample, 457 (63.3%) were male; 447 (62.0%) were younger than 12 months, 207 (28.7%) were 12 to 24 months, and 68 (9.3%) were 25 to 35 months. Approximately 55.0% (n = 397) spoke English as primary language. Mean (SD) baseline functional performance skills (ie, COS scores, n = 448) were 4.6 (1.8) for social emotional skills, 3.7 (1.5) for cognitive function, and 4.3 (1.7) for adaptive and/or behavioral function (Table 1).

**Table 1.** Characteristics of 722 Low-Income Children With Developmental Disabilities and Delays Receiving Care From a Large Safety-Net Health System and EI Program

Child Characteristics	Referred and Received EI Care Plan, No. (%)
Condition type	
Developmental delay	633 (87.7)
Diagnosed condition	89 (12.3)
Special health care needs group^a^	
No special health care needs	472 (70.6)
Mild	68 (10.2)
Mild condition severity	74 (11.1)
Severe	55 (8.2)
Birth weight by category, kg	
<1.5	25 (3.5)
1.5-2.5	61 (8.5)
>2.5	636 (88.1)
Child’s age, mo	
<12	447 (62.0)
12-24	207 (28.7)
25-35	68 (9.3)
Family annual income, $	
<20 000	663 (91.8)
≥20 000	59 (8.2)
Race/ethnicity	
White, non-Hispanic	105 (14.5)
Black, non-Hispanic	67 (9.3)
Hispanic	532 (73.7)
Other, non-Hispanic	18 (2.5)
Primary language	
English	397 (55.0)
Other	325 (45.0)
Female	265 (36.7)
Entry Child Outcome Summary subscale scores, mean (SD) (n = 448)^b^	
Positive social emotional skills	4.6 (1.8)
Acquiring and using knowledge and skills	3.7 (1.5)
Taking appropriate action to meet needs	4.3 (1.7)

Abbreviation: EI, early intervention.

^a^ Fifty-three children were missing special health care needs information, so the sample size was 669.

^b^ Each Child Outcome Summary subscale score was derived from health care professional clinical judgment, parent concerns, and developmental assessment results and measured on a 7-point scale, from 1, very early skills (ie, child does not use any immediate foundational skills related to this outcome) to 7, all skills expected (ie, there are no concerns about the child’s function in this area).

### Service Use Characteristics

The median (interquartile range [IQR]) for service use characteristics is presented. The median number of days to receive an EI care plan was 56.0 (IQR, 1.0-111.0). Only 43% (n = 305) of sample children received an EI care plan within the 45-day deadline. The median (IQR) for all services per child EI service intensity (n = 525) was 2.7 (2.3-3.6) hours per month and ranged from 1.0 (1.0-2.7) hours per month for DI to 4.2 (3.9-4.5) hours per month for ST (Table 2).

**Table 2.** Early Intervention Service Use Characteristics Among 722 Low-Income Children Receiving Care From a Safety-Net Health System and EI Program

Characteristic	Mean (SD)	Median (IQR)
Received EI care plan in ≤45 d, No. (%)	305 (42.5)	
Time to receive EI care plan, d	105.3 (153.0)	56.0 (1.0-111.0)
Total per-child EI service intensity conditional on any use, h/mo (n = 525)^a^		
All services	3.2 (7.7)	2.7 (2.3-3.6)
Physical therapy	3.4 (1.3)	3.8 (2.5-4.3)
Occupational therapy	2.9 (1.8)	3.1 (1.90-4.28)
Speech and language pathology	4.4 (8.8)	4.2 (3.9-4.5)
Developmental intervention	1.8 (1.4)	1.0 (1.0-2.7)
Length of EI services, d (n = 640)	153.5 (97.2)	163.0 (107.0-182.0)

Abbreviations: EI, early intervention; IQR, interquartile range.

^a^ Subsample (n = 525) of sample children who initiated EI services.

### Service Use Timeliness

Compared with children without a special health care need, children with greatest condition severity received an EI care plan 54 days sooner (b, −53.5; 95% CI, −77.4 to −29.6). Compared with infants, 2-year-old children received an EI care plan 52 days sooner (b, −52.0; 95% CI, −79.7 to −24.3). Finally, children from a race and ethnicity categorized as other, non-Hispanic received an EI care plan 43 days sooner (b, −42.5; 95% CI, −65.5 to −19.5) than their white, non-Hispanic counterparts (Table 3).

**Table 3.** Adjusted Quantile Median Regression Models Estimating the Number of Days Between Early Intervention Referral and Receipt of an Early Intervention Care Plan^a^

Independent Variables	b (95% CI)
Condition type	
Diagnosed condition	2.5 (−17.6 to 22.6)
Special health care needs group	
No special health care needs	[Reference]
Mild condition severity	−3.5 (−20.9 to 13.9)
Moderate condition severity	−1.5 (−23.3 to 20.3)
Greatest condition severity	−53.5 (−77.4 to −29.6)^b^
Birth weight by category, kg	
<1.5	[Reference]
1.5-2.5	9.5 (−37.2 to 56.2)
>2.5	−7.0 (−32.0 to 18.0)
Child’s age, mo	
<12	[Reference]
12-24	−6.5 (−19.6 to 6.6)
25-35	−52.0 (−79.7 to −24.3)^b^
Family income (annually), $	
≥20 000	−2.5 (−17.1 to 12.1)
Race/ethnicity	
White, non-Hispanic	[Reference]
Black, non-Hispanic	1.5 (−23.1 to 26.1)
Hispanic	9.5 (−7.1 to 26.1)
Other, non-Hispanic	−42.5 (−65.5 to −19.5)^b^
Primary language, English	1.5 (−11.3 to 14.3)
Female child	5.5 (−7.0 to 18.0)

^a^ Results are based on a subsample of 663 children who had complete information for these variables.

^b^ *P* < .001.

### Service Intensity

Compared with children with no special health care need, children with a condition of mild severity received more intensive physical therapy (b, 0.9; 95% CI, 0.1-1.6). Children with a condition of moderate severity received more intensive DI (b, 0.9; 95% CI, 0.3-1.4). Compared with infants born less than 1.5 kg, babies with a birth weight greater than 2.5 kg received more intensive DI (b, 0.7; 95% CI, 0.1-1.3). Compared with infants, 1-year-old children received more intensive DI (b, 0.5; 95% CI, 0.1-1.0) than children with no special health care need and 2-year-old children received less intensive OT (b, −1.0; 95% CI, −1.9 to −0.03). Children whose family income was above the FPL received more intensive OT (b, 1.9; 95% CI, 0.9-3.0), whereas children from a race and ethnicity categorized as other, non-Hispanic received less intensive OT (b, −1.6; 95% CI, −2.6 to −0.7) (Table 4).

**Table 4.** Adjusted Quantile Median Regression Models Estimating Early Intervention Service Use Intensity Overall and for Each Core Early Intervention Service

Independent Variables	Intensity, b (95% CI)				
	Total	PT	OT	ST	DI
No. of observations	525	109	89	396	265
Condition type					
Diagnosed condition	−0.6 (−1.3 to 0.1)	0.2 (−0.6 to 1.1)	0.1 (−0.6 to 0.9)	−0.5 (−1.6 to 0.5)	−0.4 (−0.9 to 0.1)
Special health care need group					
No special health care need	[Reference]	[Reference]	[Reference]	[Reference]	[Reference]
Mild condition severity	−0.4 (−1.3 to 0.6)	0.9 (0.1 to 1.6)^a^	−0.5 (−1.6 to 0.5)	−0.7 (−1.7 to 0.4)	0.4 (−0.39 to 1.1)
Moderate condition severity	−0.2 (−0.8 to 0.5)	−0.8 (−1.8 to 0.1)	−0.5 (−1.6 to 0.5)	−0.3 (−1.2 to 0.6)	0.9 (0.3 to 1.4)^b^
Greatest condition severity	0.01 (−0.6 to 0.6)	0.1 (−0.9 to 1.0)	0.8 (−0.2 to 1.9)	−0.3 (−1.6 to 1.0)	−0.2 (−0.8 to 0.3)
Birth weight by category, kg					
<1.5	[Reference]	[Reference]	[Reference]	[Reference]	[Reference]
1.5-2.5	0.1 (−0.6 to 0.7)	0.4 (−0.8 to 1.5)	0.7 (−0.8 to 2.1)	0.5 (−0.6 to 1.6)	0.3 (−0.4 to 1.0)
>2.5	0.9 (−0.04 to 1.8)	0.7 (−0.2 to 1.5)	−0.3 (−1.3 to 0.8)	1.1 (−0.3 to 2.4)	0.7 (0.1 to 1.3)^a^
Child’s age, mo					
<12	[Reference]	[Reference]	[Reference]	[Reference]	[Reference]
12-24	−0.2 (−1.2 to 0.9)	0.4 (−0.2 to 1.0)	0.6 (−0.2 to 1.3)	−0.6 (−2.2 to 0.9)	0.5 (0.1 to 1.0)^a^
25-35	−0.4 (−1.44 to 0.7)	−0.8 (−1.6 to 0.03)	−1.0 (−1.9 to −0.03)^a^	−0.1 (−1.8 to 1.5)	−0.2 (−0.9 to 0.5)
Family income (annually), $					
≥20 000	−0.3 (−0.8 to 0.3)	−0.6 (−1.5 to 0.3)	1.9 (0.9 to 3.0)^c^	−0.2 (−0.9 to 0.6)	−0.1 (−0.6 to 0.5)
Race/Ethnicity					
White, non-Hispanic	[Reference]	[Reference]	[Reference]	[Reference]	[Reference]
Black, non-Hispanic	0.03 (−0.4 to 0.5)	−1.0 (−2.4 to 0.4)	1.2 (−0.1 to 2.4)	0.2 (−0.5 to 1.0)	0.1 (−0.6 to 0.7)
Hispanic	0.9 (−0.9 to 2.7)	−0.1 (−0.8 to 0.6)	−0.1 (−1.0 to 0.8)	1.1 (−1.4 to 3.6)	0.04 (−0.4 to 0.5)
Other, non-Hispanic	0.7 (−0.3 to 1.8)	−0.02 (−0.9 to 0.9)	−1.6 (−2.6 to −0.7)^c^	0.3 (−1.3 to 1.8)	0.7 (−0.3 to 1.7)
Primary language, English	0.7 (−1.1 to 2.5)	−0.4 (−0.9 to 0.1)	0.1 (−0.8 to 0.9)	1.0 (−1.6 to 3.7)	−0.04 (−0.4 to 0.3)
Female child	0.9 (−0.7 to 2.6)	0.2 (−0.4 to 0.7)	−0.2 (−0.8 to 0.5)	1.2 (−1.1 to 3.5)	−0.3 (−0.7 to 0.04)

Abbreviations: DI, developmental intervention; OT, occupational therapy; PT, physical therapy; ST, speech therapy.

^a^ *P* < .05.

^b^ *P* < .01.

^c^ *P* < .001.

### Functional Outcome Analyses

An additional hour per month of all EI services was associated with a 3-point gain in functional outcomes scores (b, 3.0; 95% CI, 1.5-5.9) among children with complete outcomes information (n = 448) and this was consistent across all 3 subscales, a clinically meaningful (ie, 0.6 SD) gain. For example, a 3-point change corresponds with improvement from a score of 3 (eg, child does not yet show functioning expected of a child of his or her age in any situation) to a 6 (eg, child’s functioning generally is considered appropriate for his or her age, but there are some significant concerns about the child’s functioning and/or may border on not keeping pace with age expectations).^9^ Moreover, an additional hour per month of DI was associated with a 1.6-point gain (difference, 1.6; 95% CI, 1.2-2.1) in social-emotional function scores. An additional hour per month of ST was associated with a nearly 1-point gain (difference, 0.9; 95% CI, 0.74-1.0) in taking appropriate action to meet needs (Table 5). The validity of the COS has been reported^10^ and a 1-point gain in function corresponds with the difference between showing no age-appropriate functional skills (COS score: 4) vs showing age-appropriate skills occasionally (COS score: 5). Thus, a 1-point gain in a COS score could be a substantively meaningful difference for parents and clinicians. The use of the COS score in EI outcomes research is still novel and more research is needed to demonstrate its clinical use.

**Table 5.** Adjusted Linear Regression Estimating the Association Between Each Child Outcomes Summary Function Subscale and Early Intervention Service Intensity Overall and for Each Core Early Intervention Service^a^

Independent Variables	Difference (95% CI)		
	Positive Social-Emotional Skills	Acquiring and Using Knowledge and Skills	Taking Appropriate Action to Meet Needs
Log-transformed intensity type			
Total intensity	3.0 (1.5-5.9)^b^	3.3 (1.8-6.1)^c^	3.1 (1.5-6.5)^b^
Physical therapy intensity	1.1 (0.8-1.5)	1.2 (0.9-1.6)	1.3 (0.9-1.9)
Occupational therapy intensity	1.1 (0.9-1.3)	1.1 (0.8-1.4)	1.2 (0.9-1.6)
Speech and language pathology intensity	1.0 (0.9-1.1)	1.0 (0.9-1.2)	0.9 (0.7-1.0)^d^
Developmental intervention intensity	1.6 (1.2-2.1)^b^	1.4 (1.0-1.9)	1.2 (0.9-1.6)

^a^ Results are based on a sample of 448 children who had complete functional outcomes data.

^b^ *P* < .01.

^c^ *P* < .001.

^d^ *P* < .05.

## Discussion

The timeliness and intensity of services received in addition to the association between EI service use and changes in children’s functional outcomes among a sample of EI-referred low-income infants and toddlers with developmental delays and disabilities from a large metropolitan area were evaluated. Overall, for half the sample children, the time to receive an EI care plan was nearly 2 months, with only 43% of children in the sample receiving an EI care plan within the mandated 45-day deadline. Moreover, we found that greater EI service intensity was associated with gains in social-emotional, cognitive, and adaptive and/or behavioral function.

Regarding EI timeliness, condition severity and age appear to be important factors. Children with conditions associated with greatest severity received an EI care plan almost 2 months sooner than their peers without special health care needs. Some previous research suggests that children with more complex clinical needs face greater barriers in accessing therapy,^11,12^ yet other literature suggests that pediatricians are more likely to refer young children whose developmental trajectory is clear and concerning.^13^ Our results that children with the greatest need receive more timely services speak to the success of systems that efficiently identify and refer EI-eligible children. In the study health system, children with the most severe conditions receive additional support services (eg, family navigator), which may facilitate more timely EI access.

We also found that children aged 2 years experienced more timely receipt of an EI care plan than infants. Children aged 2 years are closer to aging out of EI than their younger counterparts, so it is encouraging that older children receive more prompt service initiation. On the other hand, infants may receive delayed care. To this end, previous literature suggests that for many high-risk infants, initiating therapy services takes 3 to 5 months.^14,15^ Future research should explore family and systems-level EI service initiation barriers to ensure equitable and seamless EI system navigation.

This study explored an additional EI care quality metric, service intensity, and found low rates. Our findings are consistent with previous research suggesting that EI intensity falls well below therapeutic levels, even after adjusting for varied clinical needs.^16^ Moreover, among a nationally representative sample of EI enrolled children, most children receive less than 2 hours of EI per week^17^ with more recent research suggesting service intensities of less than 1 hour per week.^18^ In the current study, children received about 45 minutes of EI services per week, ranging from 15 minutes of DI to more than 1 hour of ST weekly. We acknowledge that EI service delivery models encourage parent support and education to implement intervention strategies within the child’s typical routine. While this model decreases the need for greater amounts of direct clinician intervention, 14% of EI parents nationally report unmet therapy needs.^17^ Our findings suggest a need to further explore barriers to optimal EI therapy intensity.

The finding that children from families above the FPL received more intensive OT is consistent with previous literature.^18^ Although the current study cannot determine mechanisms to explain income disparities, previous literature suggests a lack of communication and coordination, particularly between the EI and medical systems.^13,19^ Moreover, parents often struggle to understand the role of EI.^20^ Thus, families with higher incomes may have greater resources to overcome these barriers and advocate for more intensive therapy.

We also found that greater EI service intensity was associated with larger gains in function. To our knowledge, this is the first published study to leverage outcomes data linked with service use, and adjust for varying child characteristics to determine associations between EI services and meaningful outcomes. Although these data are a promising first step in describing high-value EI, we acknowledge that these associations are not causal and that families who participate in EI may differ from families who cannot, particularly on characteristics related to developmental gains. Thus, robust pragmatic trials of different EI service models are needed to show EI effectiveness.

### Limitations and Strengths 

This study had limitations. First, this study included 1 health system and 1 EI program, which limits the generalizability of the results. In particular, the sample was drawn from a safety-net health system that serves low-income families. However, most EI programs serve diverse families where young children have experienced numerous adverse childhood events (eg, homelessness, abuse, and neglect) and the findings of this study are applicable to a subset of children served by all EI programs. Moreover, we leveraged the overlapping catchment areas of health and EI systems, which could serve as a framework for similar coordination in other geographical areas where federally qualified health centers and EI programs serve a similar catchment area. Second, service use information was available for about three-fourths of referred children and of those, 85% had complete outcomes data. There is a potential for bias if these subsamples differed from other EI-referred children. While the groups did not differ on measured child characteristics, the possibility exists that they differed on unmeasured factors that could influence service use intensity (eg, adherence, shared decision making). Also, evaluation of care quality measures is limited to those routinely collected. We acknowledge that these measures do not fully capture the breadth of quality measures that are meaningful to EI parents and clinicians.

Despite the limitations, this study had a number of strengths. To our knowledge, this is the first published study to follow EI-referred children to examine timeliness and service intensity. The findings suggest more than half of study children received delayed and low-intensity EI care, suggesting areas for quality improvement. We also linked pediatric primary care and EI program data, which allowed us to follow children through EI care planning and service provision and leverage existing data elements. This data system integration could serve as a framework for other institutions to track EI-referred children and build information technology infrastructure to link EI needs, services, and outcomes. Finally, these findings contribute to the limited extant literature examining EI service use and outcomes, which is a critical first step to describing high-value EI.

## Conclusions

More than half of the children in this study received delayed care, that is, greater than 56 days to EI service initiation. However, it is encouraging that children with high need and who are closer to aging out of EI received timely services. We found low rates of service use intensity overall and for core EI services. Early intervention service use intensity related to children’s age, family income, and functional gains. The findings of this study could serve as a framework for systems collaboration between pediatric primary care and EI and for measuring key EI care quality metrics together.

## Notes

Supplement. **eFigure.** Sample Selection Diagram
