# Supplementary material for: Timing and Intensity of Early Intervention Service Use and Outcomes Among a Safety-Net Population of Children
Source: JAMA Netw Open. 2019 Jan 25;2(1):e187529. doi: 10.1001/jamanetworkopen.2018.7529 (PMC6484581; doi:10.1001/jamanetworkopen.2018.7529)
Supplement: Supplement. — eFigure. Sample Selection Diagram [file jamanetwopen-2-e187529-s001.pdf]

Supplementary Online Content

McManus BM, Richardson Z, Schenkman M, Murphy N, Morrato EH. Timing and intensity of early intervention service use and outcomes among a safety-net population of children. *JAMA Netw Open*. 2019;2(1):e187529. doi:10.1001/jamanetworkopen.2018.7529

eFigure. Sample Selection Diagram

This supplementary material has been provided by the authors to give readers additional information about their work

© 2019 McManus BM. *JAMA Network Open*.

eFigure. Sample Selection Diagram

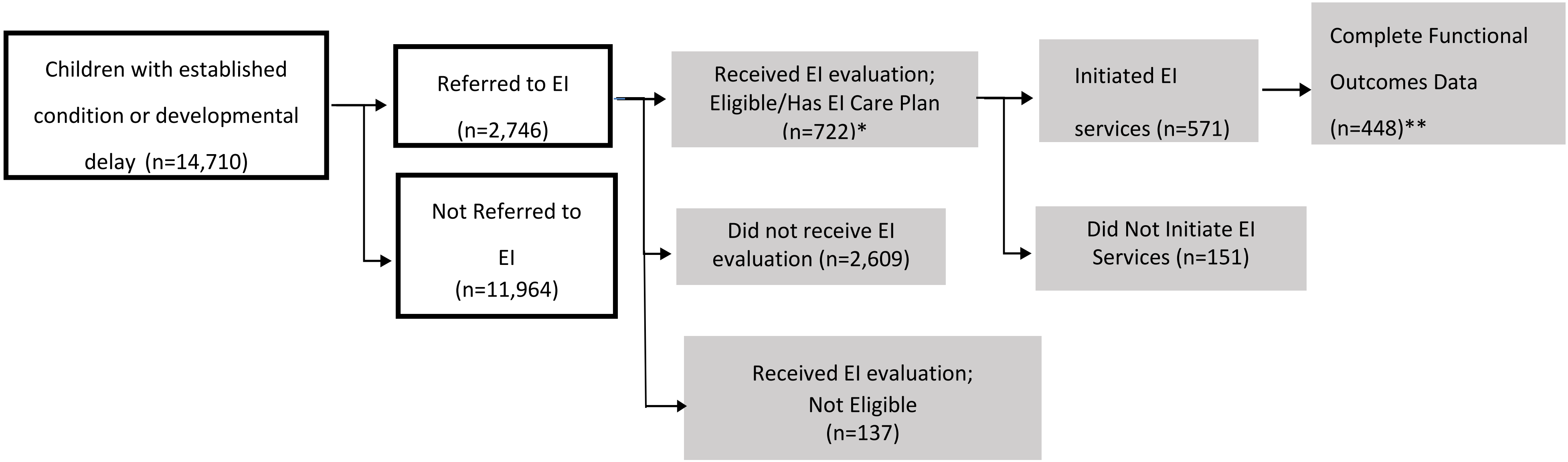

☐ Collected from Denver Health electronic health record, 10/1/14-6/30/16

Collected from Rocky Mountain Human Services EI program EI record, 10/1/14- 9/30/16

*Analytic Sample: Intensity Outcome

**Analytic Sample: Functional Outcomes

© 2019 McManus BM. *JAMA Network Open*.

© 2019 McManus BM. *JAMA Network Open*.
